# Synthesis and Application of a 2-[(4-Fluorophenyl)-sulfonyl]ethoxy Carbonyl (Fsec) Protected Glycosyl Donor in Carbohydrate Chemistry 

**DOI:** 10.3390/molecules15085708

**Published:** 2010-08-19

**Authors:** Sara Spjut, Weixing Qian, Mikael Elofsson

**Affiliations:** Department of Chemistry, Umeå University, SE-90187 Umeå, Sweden

**Keywords:** base sensitive protecting group, *O*-protection, glycoconjugate synthesis, glycosylation

## Abstract

The 2-[(4-fluorophenyl)sulfonyl]ethoxy carbonyl (Fsec) group for protection of hydroxyl groups has been designed, synthesized, and evaluated. Fsec-Cl was readily prepared in 91% yield over three steps and subsequently used to protect 4-fluorobenzyl alcohol in high yield. The Fsec group was cleaved from the resulting model compound under mild basic conditions e.g., 20% piperidine in DMF and was stable under acidic conditions, e.g., neat acetic acid. The Fsec group was used to protect the unreactive 4-OH in a galactose building block that was later used in the synthesis of 6-aminohexyl galabioside.

## 1. Introduction

**Figure 1:**
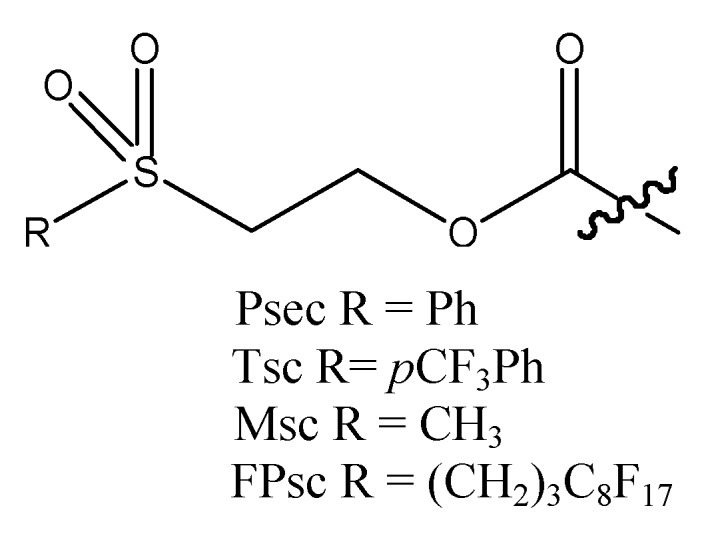
The Psec, Tsc, Msc, and FPsc protecting groups.

Regioselectivity is often a challenge in glycoconjugate synthesis since carbohydrates contain several hydroxyl groups with different reactivity. Therefore, orthogonal protecting group strategies are of great importance. Esters, ethers, and acetals are some of the most common protecting groups used for hydroxyl groups in carbohydrate chemistry and they can be quite easily introduced and later cleaved selectively [1,2]. Due to the high number of protecting group manipulations that are frequently necessary synthesis of glycoconjugates is both time consuming and cumbersome. To address this, solid-phase synthesis has been applied in order to simplify synthesis due to the fact thatonly a filtration is applied after each reaction step and more thorough purification is not performed until the final target compound has been cleaved from the resin [3]. The 9*H*-fluoren-9-yl-methoxycarbonyl (Fmoc) group is widely used as a *N*-protecting group in solid-phase synthesis of peptides [4]. The Fmoc group has also been used in some extent as an *O*-protecting group in solution and solid-phase synthesis of carbohydrates [5,6,7]. It can easily be removed under mild basic conditions (e.g., 20% triethylamine in CH_2_Cl_2_ [5]) and it is stable under acidic conditions. The Fmoc group is however lipophilic and large (and therefore sterically demanding) so alternative groups have been developed. The 2-(phenylsulfonyl)ethoxycarbonyl (Psec) group and the closely related 4-chloro-, 4-nitro-, and 4-methyl-derivatives are interesting alternatives that have been applied for *O*-protection (Figure 1) [8,9]. With more electronegative substituents in position 4 of the phenyl ring faster cleavage was observed with triethylamine (15 eq. in pyridine at room temperature). The fluorinated 2-(4-trifluoromethylphenylsulfonyl)ethoxycarbonyl (Tsc) group (Figure 1) has been used for *N*-protection of amino acids in solid-phase synthesis of polyamides [10]. The Tsc group can be removed under basic conditions, e.g., 0.1 M LiOH in THF-H_2_O (1:1) at 0 °C. The methylsulfonylethoxycarbonyl (Msc) group (Figure 1) has been synthesized and evaluated for *O*-protection in carbohydrate chemistry [11] but was originally developed and used for *N*-protection of amino acids [12]. The Msc group is an alternative to the Fmoc group since it is less lipophilic and sterically demanding. Msc protected alcohols were deprotected in quantitative yields with e.g., tetra-*N*-butylammonium fluoride (TBAF, 0.1 eq. in THF for 30 min) or 1,8-diazabicyclo[5.4.0]undec-7-ene (DBU, 0.1 eq. in DMF for 25 min) [11]. In addition the fluorous propylsulfonylethoxy carbonyl (FPsc) group (Figure 1) was synthesized and evaluated for *O*-protection in carbohydrate chemistry [11]. The FPsc group is a fluorous analog of the Msc group and was applied in synthesis of a trisaccharide exploiting the fluorous component in the FPsc group (F_17_C_8_-) for purification by fluorous solid-phase extraction [11].

To further explore the potential of solid-phase glycoconjugate synthesis our laboratory has developed gel-phase ^19^F-NMR spectroscopy as a technique to monitor the outcome of reactions while the product is still attached to the solid support [13,14,15,16,17]. Yield and diastereomeric ratio can been determined by using fluorine containing protecting groups and linkers using standard NMR spectroscopy equipment. However, in our hands Fmoc protection of hindered and unreactive alcohols has been sluggish, indicating the need for alternative protective groups. We therefore designed the 2-[(4-fluorophenyl)sulfonyl]ethoxy carbonyl chloride (Fsec-Cl) **4** suitable for *O*-protection in both solution- and solid-phase synthesis monitored with gel-phase ^19^F-NMR spectroscopy. Herein we describe the synthesis and characterization of Fsec protected alcohols and the use of a 4-*O*-Fsec protected galactose donor in solution synthesis of 6-aminohexyl galabioside.

## 2. Results and Discussion

**Scheme 1:**
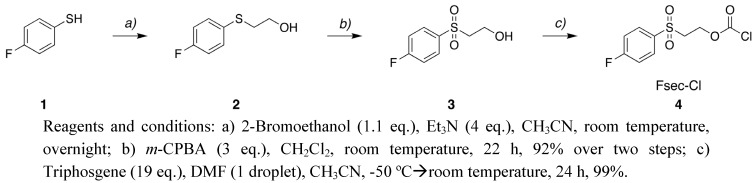
Synthesis of Fsec-Cl **4**.

Given the stability profiles and cleavage conditions for the *N*-Tsc [10,18], *O*-Psec and related derivatives [8,9], and *O*-Msc [11] groups we reasoned that the Fsec group should have properties suitable for *O*-protection of carbohydrates. The Fsec protecting group reagent 2-[(4-fluorophenyl)sulfonyl]ethoxy carbonyl chloride (Fsec-Cl) **4** was synthesized in 91% yield over three steps (Scheme 1). 4-Fluorothiophenol (**1**) was reacted with 2-bromoethanol under basic conditions to give 2-[(4-fluorophenyl)thio]ethanol (**2**). Subsequent oxidation with *m*-CPBA produced compound **3** [19] in 92% yield. Compound **3** is currently commercially available from several sources. Finally **3** was reacted with triphosgene to afford Fsec-Cl **4** in quantitative yield.

**Scheme 2:**
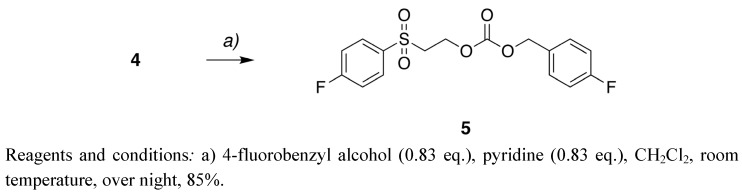
Synthesis of 4-fluorobenzyl 2-[(4-fluorobenzyl)sulfonyl]ethyl carbonate (**5**).

The stability of the Fsec protecting group was tested under both basic and acidic conditions using the model substance 4-fluorobenzyl 2-[(4-fluorobenzyl)sulfonyl]ethyl carbonate (**5**, Scheme 2). 4-Fluorobenzyl alcohol was protected with Fsec-Cl 4 under basic conditions in 85% yield (Scheme 2) indicating that *O-*protection with the Fsec group can readily be performed.

We found that the Fsec group could be effectively removed from **5 **with reagents commonly used for Fmoc deprotection, e.g., 20% piperidine or 2% DBU in DMF or CH_2_Cl_2_, and 1.1 eq. TBAF in THF at room temperature (Table 1, entry 1-3, 6, and 7). The reactions were monitored with LC-MS and the chromatograms showed no formation of by-products. In addition we found that the Fsec group is stable under acidic conditions (Table 1, entry 8-10) and this is critical since glycosylation reactions generally are performed under acidic conditions. 

**Table 1:** .Investigation of stability and suitable cleavage conditions of the Fsec group.

Entry	Reagents	Solvents	Time (min)	Yields^a^
1	1.1 eq. TBAF	THF	3	Quant.
2	20 % piperidine	DMF	3	Quant.
3	20 % piperidine	CH_2_Cl_2_	6	Quant.
4	20 % morpholine	DMF	> 60	Quant.
5	20 % morpholine	CH_2_Cl_2_	360	~ 90 %
6	2 % DBU	DMF	3	Quant.
7	2 % DBU	CH_2_Cl_2_	3	Quant.
8	5 % AcOH	THF	300	Stable
9	5 % TFA	THF	300	Stable
10	Neat AcOH	-	300	Stable

^a^ Estimated from LC-UV traces.

**Scheme 3:**
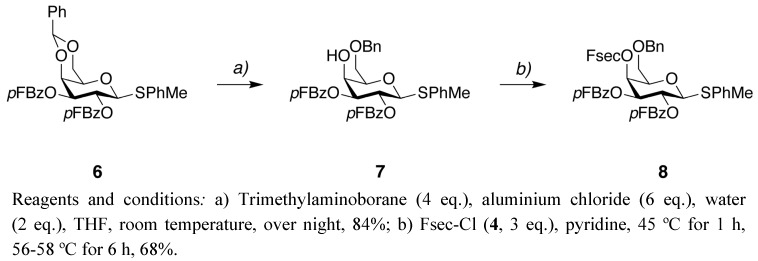
Synthesis of the Fsec protected galactose donor **8**.

Since the Fsec protecting group met our requirements concerning stability and cleavage conditions we decided to apply it for *O*-protection of a glycosyl donor. The Fsec protected glycosyl donor **8** was synthesized in two steps from the fully protected galactose donor **6** [20] (Scheme 3). Compound **6** was treated with trimethylaminoborane and aluminium chloride [21] to open the acetal ring and give **7** with a free 4-OH in 85% yield. We had earlier failed in our efforts to introduce the Fmoc group on the free 4-OH in **7**, e.g., with Fmoc-Cl and pyridine in room temperature, or Fmoc-Cl and 4-DMAP under microwave irradiation. However, when Fsec-Cl **4** was reacted with **7** in neat pyridine the expected product **8** was formed in 68% yield, indicating that the Fsec group is suitable for protection of sterically hindered and unreactive hydroxyl groups.

**Scheme 4:**
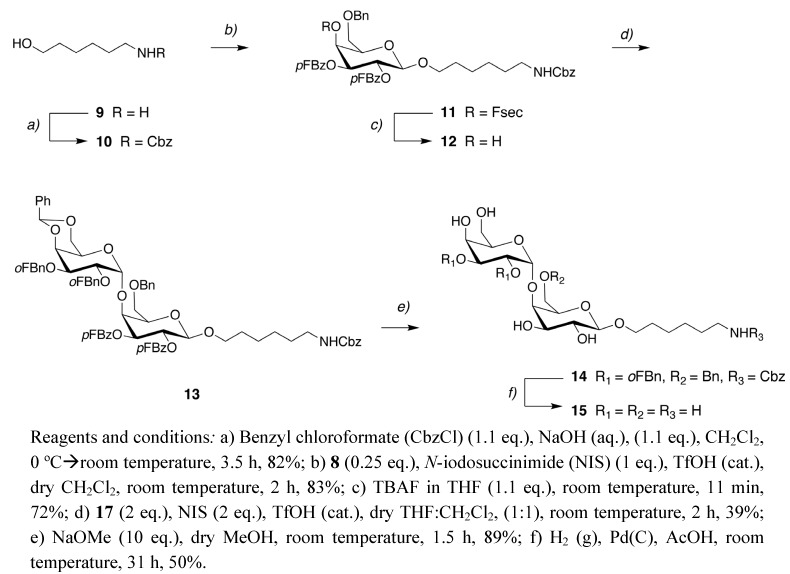
Synthesis of 6-aminohexyl galabioside **15**.

The Fsec protected galctose donor **8** was then used in synthesis of 6-aminohexyl galabioside (**15**) (Scheme 4). As a first step 6-amino-1-hexanol (**9**) was protected with benzyl chloroformate (CBz-Cl) to give the *N*-protected spacer **10 **in 82% yield. Next, the hydroxyl moiety of **10 **was glycosylated with the Fsec protected galactose donor **8** under promotion with *N*-iodosuccinimide (NIS) and trifluoro-methanesulfonic acid (TfOH) to give the β-glycoside **11** in 83% yield. The Fsec group was removed with 1.1 eq. TBAF in THF for ~10 min to furnish **12** in 72% yield. The free hydroxyl group was then glycosylated with the galactose donor **17 **(Scheme 4) under promotion with NIS and TfOH giving the fully protected galabiose derivative **13** in 39% yield. The glycosyl acceptor **12** was recovered in 40% yield, illustrating the low nucleophilicity of the 4-OH in the galactose acceptor **12**. No formation of the corresponding β-glycoside was observed. 

**Scheme 5:**
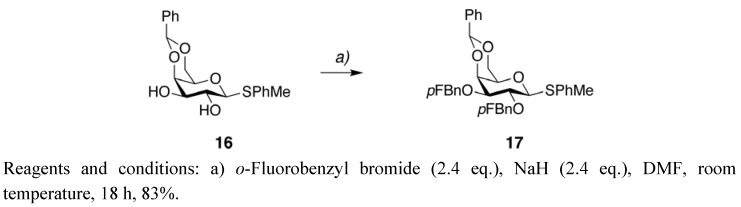
Synthesis of the galactose donor **17**.

The galactose donor **17** was formed by benzylation of **16** [15] with *o*-fluorobenzyl bromide and NaH in 83% yield (Scheme 5). The benzoyl protecting groups in **13** were removed with sodium methoxide in methanol and the remaining protecting groups were removed with palladium on charcoal in acetic acid producing the target compound **15** in 45% yield over two steps (Scheme 3). The amino group allows conjugation to a wide variety of carriers and surfaces including proteins and microwell plates [20]. 

The Fsec group has potential to be used to protect glycosyl donors intended for solid-phase synthesis of glycoconjugates using gel-phase ^19^F-NMR spectroscopy as monitoring technique. The chemical shift for the fluorine atom in the Fsec group (approximately -103 ppm) is well separated from other commonly used protecting groups, e.g., fluorine containing benzyl (-114 to -119 ppm), benzoate (-104 to -113 ppm), or benzylidene (-112 to -121 ppm) groups [15], and the recently introduced [1-(4-(4-fluorophenyl)-2,6-dioxocyclohexylidene)ethyl] (Fde) *N*-protecting group (approximately -116 ppm) [22].^19^F-NMR shifts for the fluorinated protecting groups in compound **4**-**8** and **11**-**14 **are given in Table 2. Hence, the Fsec group can likely be used in combination with these protecting groups as part of an orthogonal protecting group strategy.

**Table 2:** ^19^F-NMR shifts for the fluorinated protecting groups in compound **4**-**8** and **11**-**14**.

ID	^19^F-NMR shift *δ* [ppm]^a^		
	Fsec	FBn	FBz
**4**	-102.7		
**5**	-103.3	−113.2	
**6**			-105.2, -105.6
**7**			-105.0, -105.4
**8**	-102.8		-105.1, -104.6
**11**	-103.0		-104.6, -105.2
**12**			-105.1, -105.5
**13**		-118.7, -119.3	-104.4, -105.5
**14**		-118.9, -119.4	

^a ^Relative CFCl_3_.

## 3. Experimental

### 3.1. General

All reactions were carried out under inert atmosphere with dry solvents under anhydrous conditions, unless otherwise stated. CH_2_Cl_2_ was distilled from calcium hydride, DMF was distilled under vacuum, CH_3_CN was distilled from CaH_2_, and THF distilled from potassium. TLC was run on Silica Gel 60 F_254_ (Merck) and the spots were detected in UV-light and stained with H_2_SO_4 _in ethanol and heat. Silica gel (Matrex, 60 Å, 35–70 mm, Grace Amicon) and solvents of analytical grade were used for flash column chromatography except where otherwise is stated. In these cases, Biotage Isolera One^TM^ with SNAP KP-SIL columns (10–50 g) and n-heptane-EtOAc gradients were used for flash column chromatography, typically with a flow rate of 12–40 mL min^-1^, depending on the size of the column. ^1^H- and ^13^C-NMR spectra were recorded at 298 K on a Bruker DRX-400 instrument at 400 and 100 MHz, respectively, with CDCl_3_ or D_2_O as solvents and residual CHCl_3_ (δ_H_ 7.27 ppm) or D_2_O (δ_H_ 4.79 ppm) as internal standard for ^1^H and CDCl_3_ (δ_C_ 77.23 ppm) as internal standard for ^13^C. Peaks that could not be assigned are not reported. *J* values are given in Hz. Proton decoupled ^19^F-NMR spectra were recorded at 298 K on the Bruker DRX-400 at 376 MHz in CDCl_3_ with CFCl_3_ (δ_F_0.00 ppm) as internal standard. Positive and negative electrospray mass analyses were carried out on a Waters Micromass ZG 2000. Preparative HPLC separations were performed on a Beckman System Gold HPLC, using a Supelco Discovery Biowide Pore C_18_ column (250 × 212 mm, 5 μm) eluted with a linear gradient of CH_3_CN in water, both of which contained trifluoroacetic acid (0.1%). The flow rate was 11 mL min^-1^ and detection at 214 nm. Analytical HPLC were performed on a Beckman System Gold HPLC, using a Supelco Discovery Biowide Pore C_18_ column (250 × 46 mm, 5 μm) with a flow rate of 1.5 mL min^-1^ and detection at 214 nm.

*2-[(4-Fluorophenyl)thio]ethanol *(**2**). 2-Bromoethanol (2.72 g, 21.8 mmol) was slowly added to a mixture of 4-fluorothiophenol (**1**, 2.56 g, 20.0 mmol) and triethylamine (11.1 mL, 80 mmol) in CH_3_CN (50 mL) at room temperature. The reaction mixture was stirred over night and the solvent was removed under reduced pressure. To the residue diethyl ether was added to precipitate the bromide salt. The salt was filtrated by suction and washed twice with diethyl ether. The filtration was concentrated to give **2** as a yellow oil (3.82 g) that was used without further purification. ^1^H-NMR (CDCl_3_) δ 7.43–7.35 (m, 2H, Ar-H), 7.04–6.95 (m, 2H, Ar-H), 3.70 (t, 2H, *J* = 6.0 Hz, -SCH_2_C*H*_2_OH), 3.04 (t, 2H, *J* = 6.0 Hz, -SC*H*_2_CH_2_OH); ^19^F-NMR (CDCl_3_) δ -115.1; MS(ES^+^) calculated for C_8_H_10_FOS (M+H^+^) 173.04, found 173.08.

*2-[(4-Fluorophenyl)sulfonyl]ethanol* (**3**) [19]. *m*-Chloroperbenzoic acid (8.72 g, 38.7 mmol) was slowly added to **2** (2.32 g, 13.4 mmol) in CH_2_Cl_2_ (100 mL) at 0 °C. The reaction mixture was stirred for 22 h at room temperature. The solvent was concentrated to precipitate *m*-chlorobenzoic acid, which was removed by filtration. The crude product was purified by column chromatography (30–100% EtOAc in petroleum ether) to give **3** (2.45g, 92% yield)as a white solid in. ^1^H-NMR (CDCl_3_) δ8.01–7.92 (m, 2H, Ar-H), 7.33–7.21 (m, 2H, Ar-H), 4.07–3.97(m, 2H, -SO_2_CH_2_C*H*_2_OH), 3.35 (t, 2H, *J* = 5.6 Hz, -SO_2_C*H*_2_CH_2_OH), 2.64 (br. 1H, -CH_2_O*H*); ^19^F NMR (CDCl_3_): δ -103.2; MS(ES^+^) calculated for C_8_H_10_FO_3_S (M+H^+^) 205.03, found 205.29; m.p. 60–61 °C.

*2-[(4-Fluorophenyl)sulfonyl]ethoxy carbonyl chloride* (**4**). One drop of DMF was added to triphosgene (14.9 g, 0.15 mol) in CH_3_CN (40 mL) and then the solution was cooled to -50 °C. Compound **3** (2.05 g, 10.0 mmol) in CH_3_CN (50 mL) was slowly added to the reaction mixture and then stirred for 24 h at room temperature. Residual phosgene and hydrogen chloride were removed by purging with nitrogen gas for 40 min after which time the solvent was evaporated *in vacuo* to give **4** (2.65 g) as a white solid in quantitative yield. ^1^H-NMR (CDCl_3_) δ 8.00–7.91 (m, 2H, Ar-H), 7.33–7.23 (m, 2H, Ar-H), 4.65 (t, 2H, *J* = 5.6 Hz, -SO_2_CH_2_C*H*_2_O-), 3.53 (t, 2H, *J* = 5.6 Hz, -SO_2_C*H*_2_CH_2_O-);^13^C-NMR (CDCl_3_) δ 166.1 (d, *J* = 256.0 Hz), 116.9 (d, *J* = 22.5 Hz), 150.7, 135.0 (d, *J* = 3.1 Hz), 131.1 (d, *J* = 9.7 Hz), 54.3, 54.5; ^19^F-NMR (CDCl_3_): δ -102.7; m.p. 72–74 °C.

*4-Fluorobenzyl 2-[(4-fluorobenzyl)sulfonyl]ethyl carbonate* (**5**). Compound **4** (0.16 g, 0.60mmol) was added to pyridine (0.047 g, 0.60 mmol) and 4-fluorobenzyl alcohol (0.064 g, 0.50 mmol) in CH_2_Cl_2_ (2 mL). The mixture was stirred over night at room temperature. The solvent was removed under reduced pressure and the residue was purified by column chromatography (petroleum ether-EtOAc 3:1) to give **5** (0.151 g) in 85% yield.^1^H-NMR (CDCl_3_) δ 7.97–7.86 (m, 2H, Ar-H), 7.34–7.24 (m, 2H, Ar-H), 7.22–7.13 (m, 2H, Ar-H), 7.08–6.99 (m, 2H, Ar-H), 5.01 (s, 2H, -CH_2_O-), 4.46 (t, 2H, *J* = 6.0 Hz, -SO_2_CH_2_C*H*_2_O-), 3.48 (t, 2H, *J* = 6.0 Hz, -SO_2_C*H*_2_CH_2_O-); ^13^C-NMR (CDCl_3_) δ 166.0 (d, *J* = 255.6 Hz), 163.0 (d, *J* = 246.0 Hz), 154.2, 135.4 (d, *J* = 3.3 Hz), 131.2 (d, *J* = 9.8 Hz), 130.7 (d, *J* = 3.3 Hz), 130.5 (d, *J* = 8.4 Hz), 116.7 (d, *J* = 22.6 Hz), 115.7 (d, *J* = 21.4 Hz), 69.3, 69.3, 55.1; ^19^F-NMR (CDCl_3_): δ -113.2, -103.3; MS(ES^−^) calculated for C_17_H_15_F_2_O_7_S (M+HCO_2_^−^) 401.05, found 401.12.

*4-Methylphenyl 2,3-di-O-(4-fluorobenzoyl)-6-O-benzyl-1-thio-β-D-galactopyranoside* (**7**). Trimethyl-aminoborane (0.238 g, 3.26 mmol) was added to **6** [20] (0.500 g, 0.808 mmol) in distilled THF (25 mL) followed by aluminium chloride (0.639 g, 4.79 mmol). After dissolution of the reagents water (0.029 mL, 1.61 mmol) was added and the mixture was stirred at room temperature overnight. Water and HCl (1M) was added to terminate the reaction and the mixture was extracted with EtOAc. The organic phase was washed with brine, dried with MgSO_4_, and concentrated. Purification with flash column chromatography (Isolera, SNAP KP-SIL, 5–100% EtOAc in n-heptane, 22 min) gave **7** (0.419 g) in 84% yield.^1^H-NMR (CDCl_3_) δ 8.06–7.93 (m, 4H, ArH), 7.45 (d, 2H, *J* = 8.0 Hz, ArH), 7.40–7.28 (m, 5H, ArH), 7.12–6.93 (m, 6H, ArH), 5.79 (t, 1H, *J* = 9.9 Hz, H-2), 5.34 (dd, 1H, *J* = 2.9 and 9.9 Hz, H-3), 4.93 (d, 1H, *J* = 9.9 Hz, H-1), 4.61 (s, 2H, -OC*H*_2_C-), 4.43 (s, 1H, H-4), 3.96–4.83 (m, 3H, H-5 and H-6), 2.32 (s, 3H, -C*H*_3_); ^13^C-NMR (CDCl_3_) δ 167.1 (*J* = 6.7 Hz), 164.6 (*J* = 6.5 Hz), 167.5 (*J* = 54.9 Hz), 137.9 (*J* = 68.0 Hz), 133.2, 132.3 (*J* = 10.7 Hz), 129.6, 128.4, 128.3, 127.8, 127.7, 125.5 (*J* = 37.6 Hz), 125.4 (*J* = 37.6 Hz), 115.5 (*J* = 20.3 Hz), 115.6 (*J* = 20.3 Hz), 86.6, 77.2, 75.7, 73.7, 69.6, 68.3, 68.2, 21.1; ^19^F-NMR (CDCl_3_) δ -105.0, -105.4; MS(ES+) calculated for C_34_H_31_F_2_O_7_S (M+H) 621.18, found 620.99.

*4-Methylphenyl6-O-benzyl-2,3-di-O-(4-fluorobenzoyl)-4-O-[2-(4-fluorophenyl)sulfonylethoxy-carbonyl]-1-thio-β-D-galactopyranoside* (**8**). Compound **4** (0.08 g, 0.30 mmol) was added to **7** (0.062 g, 0.10 mmol) in pyridine (1.0 mL). The mixture was stirred at 45 °C for 1 h and then at 56–58 °C for 6 h. The pyridine was removed under reduced pressure and the residue was purified by column chromatography (toluene-EtOAc 3:1) to give **8** (0.058 g) as a white solid in 68% yield. ^1^H- NMR (CDCl_3_) δ 7.95–8.02 (m, 2H, Ar-H), 7.84–7.94 (m, 4H, Ar-H), 7.27–7.40 (m, 8H, Ar-H), 6.97–7.11 (m, 6H, Ar-H), 5.59 (t, 1H, *J* = 10.0 Hz, H-2), 5.48 (m, 1H, H-4), 5.39 (dd, 1H, *J* = 3.6 and 10.0 Hz, H-3), 4.87 (d, 1H, *J* = 10.0 Hz, H-1), 4.51 (dd, 2H, *J* = 11.6 and 26.2 Hz, -CH_2_O-), 4.28 (t, 1H, *J* = 6.4 Hz, -SO_2_CH_2_C*H*_2_O-), 3.99 (t, 1H, *J* = 6.4 Hz, H-5), 3.68 (dd, 1H, *J* = 6.0 and 9.8 Hz, H-6), 3.58 (dd, 1H, *J* = 6.8 and 9.8 Hz, H-6), 3.29–3.38 (m, 2H, -SO_2_C*H*_2_CH_2_O-), 2.32 (s, 3H, -C*H*_3_); ^13^C-NMR (CDCl_3_) δ 166.2 (*J *= 256.0 Hz), 166.1 (*J *= 254.1 Hz), 166.1 (*J *= 253.6 Hz), 164.4, 164.4, 154.1, 138.5, 137.7, 135.4 (*J* = 3.4 Hz), 133.1, 132.5 (*J* = 9.3 Hz), 132.4 (*J* = 9.4 Hz), 131.2 (*J* = 9.7 Hz), 129.9, 129.9, 128.9, 128.5, 128.0, 127.9, 125.5 (*J* = 3.1 Hz), 125.1 (*J* = 3.0 Hz), 117.0 (*J* = 22.7 Hz), 115.9 (*J* = 21.9 Hz), 115.8 (*J* = 21.9 Hz), 87.4, 75.9, 73.7, 73.1, 72.6, 68.4, 67.7, 61.4, 55.0, 21.3; ^19^F- NMR (CDCl_3_) δ -105.1, -104.6, -102.8 (Fsec); MS(ES+) calculated for C_43_H_39_F_3_O_12_S_2_ (M+H_2_O) 868.18, found 868.43; [α]_D_^25 ^= 4.6° (c 1.0, CHCl_3_).

*6-Benzyloxycarbonylamino-1-hexanol *(**10**). Benzyl chloroformate (CbzCl, 0.390 mL, 2.73 mmol) was added dropwise to a solution of 6-amino-1-hexanol (**9**, 0.301 g, 2.57 mmol) in NaOH (aq.) (2.81 mL, 2.81 mmol) at 0 °C. The solution was allowed to reach room temperature, stirred for 30 min and then CH_2_Cl_2_ was added (2 mL). After stirring at room temperature for 3 h the mixture was concentrated *in vacuo*. The residue was purified with flash column chromatography (Isolera, SNAP KP-SIL, 5–95% EtOAc in n-heptane, 22 min) to give **10** (0.528 g) in 82% yield.^1^H-NMR (CDCl_3_) δ 7.38–7.27 (m, 5H, ArH), 5.08 (s, 2H, -COOC*H*_2_C-), 4.87 (s, 1H, -NH-), 3.60 (t, 2H, *J* = 6.5 Hz, -C*H*_2_OH), 3.23–3.09 (m, 2H, -C*H*_2_NH-), 1.59–1.45 (m, 4H, -C*H*_2_CH_2_OH, -C*H*_2_CH_2_NH-), 1.42–1.23 (m, 4H, 2 × -CH_2_CH_2_C*H*_2_-); ^13^C-NMR (CDCl_3_) δ 156.6, 136.7, 128.6, 128.2, 66.7, 62.2, 41.0, 32.6, 30.0, 26.4, 25.4; MS(ES+) calculated for C_14_H_22_NO (M+H) 252.16, found 252.42.

*6-(Benzyloxycarbonylamino)-hexyl 6-O-benzyl-2,3-di-O-(4-fluorobenzoyl)-4-O-[2-(4-fluorophenyl-sulfonyl)ethoxycarbonyl]-β-D-galactopyranoside* (**11**). Compound **10** (0.118 g, 0.470 mmol), **8** (0.100 g, 0.118 mmol), and NIS (0.106g, 0.471 mmol) were dried under vacuum and in the absence of light for 2 h. Distilled CH_2_Cl_2_ (5 mL) and TfOH (0.1 M in CH_2_Cl_2_, 0.001 mmol) were added and the solution was stirred in room temperature in the absence of light for 2 h. The reaction mixture was concentrated and the residue was purified with flash column chromatography (Isolera, SNAP KP-SIL, 5–95% EtOAc in n-heptane, 22 min) giving **11** (0.064 g) in 83% yield.^1^H-NMR (CDCl_3_) δ 8.02–7.86 (m, 6H, ArH), 7.39–7.24 (m, 12H, ArH), 7.11–6.99 (m, 4H, ArH), 5.56 (dd, 1H, *J* = 8.0 and 10.3 Hz, H-2), 5.44 (d, 1H, *J* = 3.3 Hz, H-4), 5.37 (dd, 1H, *J* = 3.3 and 10.3 Hz, H-3), 5.09 (s, 2H, -COOCH_2_Ph), 4.65 (d, 1H, *J* = 8.0 Hz, H-1), 4.51 (q, 2H, *J* = 11.9 and 26.7 Hz, -OCH_2_Ph), 4.29 (t, 2H, *J* = 5.7 Hz, -COOCH_2_CH_2_-), 3.96 (t, 1H, *J* = 6.5 Hz, H-5), 3.92–3.85 (m, 1H, -OCHHCH_2_CH_2_-), 3.67–3.62 (m, 1H, H-6), 3.60–3.53 (m, 1H, H-6), 3.52–3.44 (m, 1H, -OCHHCH_2_CH_2_-), 3.39–3.30 (m, 2H, -COOCH_2_CH_2_-), 3.10–3.01 (m, 2H, -NHCH_2_-), 1.58–1.44 (m, 2H, -OCH_2_CH_2_CH_2_-), 1.35–1.13 (m, 6H, -CH_2_CH_2_CH_2_NH-); ^13^C-NMR (CDCl_3_) δ 167.4 (*J* = 8.6 Hz), 167.2, 164.9 (*J* = 6.5 Hz), 164.7, 164.4, 164.3, 156.4, 154.0, 137.6, 136.7, 135.3 (*J* = 5.3 Hz), 132.3 (*J* = 4.2 and 9.2 Hz), 131.2 (*J* = 9.8 Hz), 128.5 (*J* = 7.2 Hz), 128.2, 127.9, 127.8, 125.6 (*J* = 4.7 Hz), 125.1 (*J* = 4.3 Hz), 117.0, 116.8, 115.7 (*J* = 15.9 and 22.1 Hz), 101.2 (C-1), 77.2, 73.6, 72.3, 71.9, 71.8, 69.9, 69.7, 67.4, 66.7, 61.3, 54.9, 40.9, 29.7, 29.3, 26.8, 25.5; ^19^F-NMR (CDCl_3_) δ -103.0, -104.6, -105.2; MS(ES+) calculated for C_50_H_51_F_3_NO_14_S (M+H) 978.30, found 978.46.

*6-(Benzyloxycarbonylamino)hexyl 6-O-benzyl-2,3-di-O-(4-fluorobenzoyl)-β-D-galactopyranoside* (**12**). Compound **11** (0.013 g, 0.013 mmol) was dissolved in THF (13 mL) and TBAF in THF (0.015 mL, 0.015 mmol) was added. The solution was stirred at room temperature for 11 min and purification by preparative HPLC (30–100% CH_3_CN in H_2_O, 50 min) afforded **12** (7.2 mg) in 72% yield.^1^H-NMR (CDCl_3_) δ 8.07–7.96 (m, 4H, ArH), 7.38–7.28 (m, 10H, ArH), 7.08–6.99 (m, 4H, ArH), 5.72 (dd, 1H, *J* = 8.0 and 10.3 Hz, H-2), 5.25 (dd, 1H, *J* = 3.1 and 10.3 Hz, H-3), 5.09 (s, 2H, -COOC*H*_2_), 4.72 (t, 1H, *J* = 5.8 Hz), 4.63 (d, 1H, *J* = 8.0 Hz, H-1), 4.61 (d, 2H, *J* = 3.1 Hz, -OC*H*_2_Ph), 4.38 (d, 1H, *J* = 2.9 Hz, H-4), 3.96–3.87 (m, 1H, -OCH*H*CH_2_-), 3.86–3.78 (m, 3H), 3.54–3.46 (m, 1H, -OC*H*HCH_2_-), 3.05 (s, 2H, -CH_2_C*H*_2_NH-), 1.60–1.41 (m, 2H, -OCH_2_C*H*_2_CH_2_-), 1.36–1.10 (m, 6H, -OCH_2_CH_2_C*H*_2_C*H*_2_C*H*_2_CH_2_NH-); ^13^C-NMR (CDCl_3_) δ 167.4 (*J *= 16.5 Hz), 164.8 (*J *= 16.4 Hz), 164.8 (*J *= 53.8 Hz), 137.5, 132.6 (*J *= 9.5 Hz), 132.4 (*J *= 9.4 Hz), 128.7, 128.3, 128.1, 125.9 (*J *= 2.8 Hz), 125.5 (*J *= 2.8 Hz), 115.8 (*J *= 22.1 Hz), 115.7 (*J *= 22.1 Hz), 101.6, 74.5, 74.0, 73.3, 70.1, 70.0, 69.4, 68.4, 66.9, 41.0, 29.7, 29.3, 26.3, 25.6; ^19^F-NMR (CDCl_3_) δ -105.1, -105.5; MS(ES+) calculated for C_41_H_44_F_2_NO_10_ (M+H) 748.29, found 748.40.

*6-(Benzyloxycarbonylamino)hexyl 6-O-benzyl-[2,3-di-O-(4-fluorobenzoyl)-]-4-O-{4,6-O-benzylidene-2,3-di-O-(2-fluorobenzyl)-α-D-galactopyranosyl}-β-D-galactopyranoside* (**13**). Compound **12** (0.159 g, 0.213 mmol), **17** (donor) (0.252 g, 0.427 mmol) and NIS (0.097 g, 0.429 mmol) were dried under vacuum and in the absence of light for 2 h. Distilled THF-CH_2_Cl_2_ (1:1, 8 mL) and TfOH (0.1 M in CH_2_Cl_2_, 0.001 mmol) was added. The reaction mixture was stirred at room temperature in absence of light for 2 h. The mixture was then concentrated and the residue was purified with preparative HPLC (20–100% CH_3_CN in H_2_O, 50 min) to give **13** (0.100 g) in 39% yield. ^1^H-NMR (CDCl_3_) δ 8.05–7.91 (m, 4H, ArH), 7.52 (t, 1H, *J* = 7.5 Hz, ArH), 7.49–7.40 (m, 3H, ArH), 7.39–7.19 (m, 15H, ArH), 7.12–6.94 (m, 8H, ArH), 5.64 (dd, 1H, *J* = 7.8 and 10.8 Hz, H-2), 5.37 (s, 1H, -CH_2_-), 5.14 (dd, 1H, *J* = 2.6 and 10.8 Hz, H-3), 5.09 (s, 2H, -COOCH_2_-), 5.06 (d, 1H, *J* = 3.3 Hz, H-1´), 4.72–4.92 (m, 4H), 4.65 (d, 1H, *J* = 7.8 Hz), 4.41 (d, 1H, *J* = 2.6 Hz, H-4), 4.33 (d, 1H, *J* = 2.6 Hz), 4.27 (d, 2H, *J* = 2.6 Hz), 4.23 (dd, 1H, *J* = 3.2 and 10.2), 4.12 (dd, 1H, *J* = 3.3 and 10.2 Hz, H-2´), 4.02–3.85 (m, 3H, -OCHHCH_2_-), 3.84 (t, 1H, *J* = 6.4 Hz), 3.65 (dd, 1H, *J* = 6.1 and 9.7 Hz), 3.55–3.46 (m, 1H, -OCHHCH_2_-), 3.37 (dd, 2H, *J* = 12.6 and 61.2 Hz, H-6´), 3.10–3.00 (m, 2H, -CH_2_CH_2_NH-), 1.62–1.44 (m, 2H, -OCH_2_CH_2_-), 1.35–1.12 (m, 6H, -OCH_2_CH_2_CH_2_CH_2_CH_2_CH_2_NH-); ^13^C-NMR (CDCl_3_) δ 167.3 (*J* = 14.2 Hz), 165.0 (*J* = 25.0 Hz), 164.8 (*J* = 21.0 Hz), 161.9 (*J* = 27.2 Hz), 159.4 (J =27.0 Hz), 156.5, 138.2, 138.0, 136.8, 132.4 (*J* = 9.3 and 15.3 Hz), 130.7 (*J* = 4.10 Hz), 130.1 (*J* = 3.4 Hz), 129.2 (*J* = 4.7 Hz), 128.9, 128.6, 128.5, 128.2, 128.1, 127.7, 126.4, 126.1, 125.9, 125.8, 125.7, 125.6 (*J* = 3.7 Hz), 124.1 (*J* = 3.5 Hz), 124.0 (*J* = 10.0 Hz), 116.0 (*J* = 23.4 Hz), 115.8 (*J* = 22.7 Hz), 115.2 (J =28.5 Hz), 101.4, 100.8, 77.3, 76.7, 76.0, 75.9, 74.6, 74.3, 74.0, 73.2, 70.1, 70.0, 69.1, 68.0, 67.9, 66.8, 64.9 (*J* = 3.9 Hz), 63.4, 40.9, 29.9, 29.4, 26.5, 25.6; ^19^F-NMR (CDCl_3_) δ -104.4, -105.5, -118.7, -119.3; [α]_D_^25 ^+87.1° (c 1.0, CH_2_Cl_2_); MS(ES+) calculated for C_68_H_68_F_4_NO_15_ (M+H) 1214.45, found 1214.85.

*6-Aminohexyl 4-O-α-D-galactopyranosyl-β-D-galactopyranoside* (**15**). Compound **13** (0.048 g, 0.040 mmol) was dissolved in dry MeOH (12 mL) and NaOMe in MeOH (0.40 mL, 0.40 mmol) was slowly added. The solution was stirred for 1.5 h in room temperature and AcOH was then added until the pH was neutral. The mixture was concentrated *in vacuo* and subsequent purification by preparative HPLC (5–100% CH_3_CN in H_2_O, 50 min) gave **14** (0.034 g) in 89% yield. Compound **14** (0.032 g, 0.033 mmol) was then dissolved in AcOH (5 mL) and Pd(C) (0.044 mg) was added. The slurry was stirred under H_2_ (g) for 31 h. The Pd catalyst was removed by filtration through Celite and the filtrate was concentrated *in vacuo*. The residue was purified by preparative HPLC (0–100% CH_3_CN in H_2_O, 50 min) to give **15 **(7.2 mg) in 50% yield.^1^H-NMR (D_2_O) δ 4.97 (d, 1H, *J* = 3.7 Hz, H-1´), 4.49 (d, 1H, *J* = 7.7 Hz, H-1), 4.41–4.35 (m, 1H), 4.04 (d, 2H, *J* = 2.6 Hz), 3.97–3.81 (m, 5H), 3.80–3.58 (m, 5H), 3.57–3.50 (m, 1H), 3.01 (t, 2H, *J* = 7.5 Hz, -C*H*_2_NH_2_), 1.73–1.61 (m, 4H, -OCH_2_C*H*_2_-, -C*H*_2_CH_2_NH_2_), 1.48–1.36 (m, 4H, -OCH_2_CH_2_C*H*_2_C*H*_2_-); ^13^C-NMR (D_2_O) δ 103.5 (C-1), 100.8 (C-1´), 77.8, 75.7, 73.1, 71.7, 71.4, 71.0, 69.8, 69.7, 69.4, 61.2, 60.8, 40.1 (-*C*H_2_NH_2_), 29.2 (-*C*H_2_CH_2_NH_2_), 27.2 (-OCH_2_*C*H_2_-), 25.9 (-*C*H_2_C H_2_CH_2_NH_2_), 25.3 (-OCH_2_CH_2_*C*H_2_-); [α]_D_^25^ +38.8° (c 1.0, H_2_O); MS(ES+) calculated for C_18_H_36_NO (M+H) 442.23, found 442.25.

*4-Methylphenyl 4,6-O-benzylidene-2,3-di-O-(2-fluorobenzyl)-1-thio-β-D-galactopyranoside *(**17**). Compound **16** [15] (0.401 g, 1.071 mmol) was dissolved in DMF (6 mL) and stirred at 0 °C for 30 min before NaH (60% oil dispersion, 2.58 mmol) was added. After 10 min 2-fluorobenzyl bromide (0.311 mL, 2.58 mmol) was added dropwise and the mixture stirred at room temperature for 18 h. The reaction was quenched with MeOH and the mixture was diluted with CH_2_Cl_2_ and washed with H_2_O. The crude was concentrated and purified with flash column chromatography (n-heptane-EtOAc 5:1) to give **17** (0.522 g) as a white solid in 83%. ^1^H-NMR (CDCl_3_) δ 7.75–7.68 (m, 2H, ArH), 7.66–7.56 (m, 2H, ArH), 7.55–7.40 (m, 4H, ArH), 7.37–7.26 (m, 2H, ArH), 7.20 (t, 1H, *J* = 7.4 Hz, ArH), 7.16–7.02 (m, 5H, ArH), 5.57 (s, 1H, PhC*H*-), 4.84 (s, 2H, *o*FPhC*H*_2_O-), 4.80 (dd, 2H, *J* = 10.8 and 126.8 Hz, *o*FPhC*H*_2_O-), 4.65 (d, 1H, *J* = 9.3 Hz, H-1), 4.44 (d, 1H, *J* = 9.3 Hz, H-6), 4.31 (d, 1H, *J* = 2.7 Hz, H-4), 4.06 (d, 1H, *J* = 12.3 Hz, H-6), 3.92 (t, 1H, *J* = 9.3 Hz, H-2), 3.71 (dd, 1H, *J* = 2.7 and 9.3 Hz, H-3), 4.46 (s, 1H, H-5), 2.28 (s, 3H, -PhC*H*_3_); ^13^C-NMR (CDCl_3_) δ 161.7 (*J* = 16.2 Hz), 159.3 (*J* = 16.9 Hz), 138.0, 137.7, 133.4, 130.6 (*J* = 4.2 Hz), 130.3 (*J* = 4.1 Hz), 129.7, 129.5 (*J* = 8.2 Hz), 129.3 (*J* = 8.0 Hz), 129.0, 128.7, 128.1, 126.7, 125.7 (*J* = 14.6 Hz), 125.1 (*J* = 14.6 Hz), 124.1 (*J* = 3.4 and 15.2 Hz), 115.1 (*J* = 21.5 Hz), 101.2, 86.5, 81.7, 75.3, 73.3, 69.8, 69.4, 68.4 (*J* = 4.0 Hz), 64.9 (*J* = 3.8 Hz), 21.2; ^19^F-NMR (CDCl_3_) δ -119.1, -119.6; MS(ES+) calculated for C_34_H_33_F_2_O_5_S 591.20 (M+H), found 591.25.

## 4. Conclusions

The 2-[(4-fluorophenyl)sulfonyl]ethoxy carbonyl (Fsec) group has been evaluated as a novel *O*-protecting group. The protective group reagent Fsec-Cl is prepared in high yield in three steps and can be used to protect unreactive and sterically hindered alcohols, e.g., the 4-OH in galactose. The Fsec group is efficiently removed under mild basic conditions e.g., 20% piperidine in DMF or 1.1 eq. TBAF in THF. In addition the group was found to be stable in 5% TFA in THF and neat acetic acid. A 4-*O*-Fsec protected galactose donor was prepared and successfully applied in the synthesis of 6-aminohexyl galabioside. The Fsec group can be used for solution synthesis as well as solid-phase synthesis monitored by gel-phase ^19^F-NMR spectroscopy.
